# A Systematic Review of Perinuclear Antineutrophil Cytoplasmic Antibody-Associated Glomerulonephritis Following Coronavirus Disease 2019 Vaccination: A 2024 Update

**DOI:** 10.7759/cureus.59390

**Published:** 2024-04-30

**Authors:** Ikponmwosa J Ogieuhi, FNU Suman, Nikita Kumari, Bai Manita, Dinkey Kumari, Joti Devi, Mohamed Abdalla, Eithar Shabbo, Utsav Patel, Iqra Samreen, Khalid H Mohamed, Zahoor Ahmed, Hira Nasir

**Affiliations:** 1 Physiology, University of Benin, Benin City, NGA; 2 General Medicine, Siberian State Medical University, Tomsk, RUS; 3 Internal Medicine, Ghulam Muhammad Mahar Medical College, Sukkur, PAK; 4 Internal Medicine, Peoples University of Medical and Health Sciences, Nawabshah, PAK; 5 Pharmacy, Clifton Medical Services, Karachi, PAK; 6 Internal Medicine, Dallah Hospital, Riyadh, SAU; 7 School of Medicine, Ahfad University for Women, Omdurman, SDN; 8 Internal Medicine, Medical College, Baroda and Sir Sayaji General (SSG) Hospital, Vadodara, IND; 9 Medical School, Deccan College of Medical Sciences, Hyderabad, IND; 10 Neurology, Sheffield Teaching Hospitals NHS Foundation Trust, Sheffield, GBR; 11 Internal Medicine, Mayo Hospital, Lahore, PAK

**Keywords:** covid-19 vaccine, glomerulonephritis, mpo-anca, mrna covid-19 vaccine, p-anca

## Abstract

Antineutrophil cytoplasmic antibody (ANCA)-associated glomerulonephritis (GN) is an immune-mediated kidney disease characterized by the inflammation of small blood vessels in the kidney, leading to renal impairment and potentially irreversible damage. Concerns have been raised over the reports of myeloperoxidase/perinuclear (MPO/p) ANCA GN following the coronavirus disease 2019 (COVID-19) vaccination. Our study provides a comprehensive insight into perinuclear anti-neutrophil cytoplasmic antibodies (p-ANCA) GN after COVID-19 vaccination.

We conducted a comprehensive literature search on PubMed, Cochrane Library, and EMBASE using the Medical Subject Headings (MeSH) terms related to “covid-19 vaccine,” “glomerulonephritis,” “p-ANCA,” and “MPO-ANCA” up to March 5, 2024, to include cases of p-ANCA-associated GN following COVID-19 vaccination.

Of the 4,102 articles, we included 29, reporting 35 patients demonstrating COVID-19 vaccine-induced p-ANCA GN, with 23 (65.7%) females and a median age of 69 years (mean ± SD = 63.22 ± 16). Twenty-six (74.28%) patients received the mRNA vaccine (Pfizer = 19, Moderna = 7). Seventeen (48.57%) patients presented with p-ANCA GN after the second dose of the COVID-19 vaccine, with a median gap of 19 days (1-84 days). Constitutional symptoms (54.28%) and acute kidney injury (42.85%) were the most reported initial presentations, and elevated serum creatinine (mean peak serum creatinine = 4.98 ± 5.02 mg/dL), hematuria, and proteinuria were the laboratory findings. MPO/p-ANCA was positive in 31 (88.6%) patients. All patients underwent renal biopsy, and crescentic GN was the most common finding among 27 (77.14%) patients. Management of p-ANCA GN included steroids in 30 (85.71%) patients, followed by rituximab (28.57%), and plasmapheresis (22.86%). Most patients responded well to treatment, with complete remission in 29 (82.86%) and relapse in four (11.42%) patients. Two patients did not achieve remission and became dialysis dependent.

ANCA-associated GN is a rare and life-threatening complication of the COVID-19 vaccine, necessitating urgent evaluation and management. COVID-19 vaccine-induced p-ANCA GN should be included in the differential diagnoses of patients presenting with kidney injury after vaccination.

## Introduction and background

The rapid development and deployment of the coronavirus disease 2019 (COVID-19) vaccines against the severe acute respiratory syndrome coronavirus 2 (SARS-CoV-2) have represented a significant milestone in the global effort to mitigate the COVID-19 pandemic [1]. These vaccines have shown promising results in preventing severe morbidity, viral transmission, and mortality. Although COVID-19 vaccines have established efficacy, continuous evaluation and monitoring of potential adverse events related to the COVID-19 vaccine are warranted to ensure the vaccine’s safety profile. Many vaccines have been developed against COVID-19 viral components, including mRNA, protein subunits, or viral vectors (Table 1) [2,3].

**Table 1:** COVID-19 vaccine component and its type. mRNA = messenger ribonucleic acid; COVID-19 = coronavirus disease 2019

Viral component	Type of vaccine
Viral vector component	AstraZeneca
	Sputnik V
	Johnson’s Janssen
mRNA component	Pfizer-BioNTech
	Moderna
Protein subunit	Novavax

Although each vaccine has shown promising results in preventing viral transmission and reducing the severity of the disease, vaccine-associated side effects and complications have also been highlighted. The reported complications depend on the dosing and type of the COVID-19 vaccine. The most reported side effects include injection-site pain, headache, myalgia, fatigue, malaise, and flu-like symptoms (Table 2) [4].

**Table 2:** Commonly reported side effects of COVID-19 vaccines. N = number of patients; COVID-19 = coronavirus disease 2019

Side effect	Number	%
Injection-site pain	7,734	77.34
Fatigue	4,300	43.00
Myalgia	3,967	39.67
Local swelling	3,357	33.57
Headache	3,327	33.27

COVID-19 vaccine can potentially affect any body organ system, and involvement of gastrointestinal, cardiovascular, and nervous systems has also been reported [5,6]. Renal complications of the COVID-19 vaccine, including acute kidney injury (AKI), glomerulonephritis (GN), acute interstitial nephritis, minimal change disease, and renal failure, have also been published [7]. Antineutrophil cytoplasmic antibody (ANCA)-associated GN is a devastating and noteworthy adverse event of the COVID-19 vaccine that is rarely reported [8].

ANCA GN is an immune-mediated kidney disease characterized by the inflammation of small blood vessels in the kidney, leading to renal impairment and potentially irreversible damage [9]. There are two types of ANCA GN based on the staining pattern on indirect immunofluorescence microscopy: cytoplasmic ANCA (c-ANCA) and perinuclear ANCA (p-ANCA). In c-ANCA, the primary antigen target is proteinase 3 (PR3), and myeloperoxidase (MPO) is the main antigen target in p-ANCA. COVID-19 vaccine has been reported to induce both types of ANCA GN [10]. p-ANCA GN is commonly associated with microscopic polyangiitis, drug-induced vasculitis, and eosinophilic granulomatosis with polyangiitis. Recent reports have provided a possible link between the COVID-19 vaccine and developing p-ANCA GN [11,12]. This systematic review provides detailed insights to consolidate and analyze the evidence of p-ANCA GN as an adverse event of the COVID-19 vaccine.

## Review

Methodology

**Figure 1:**
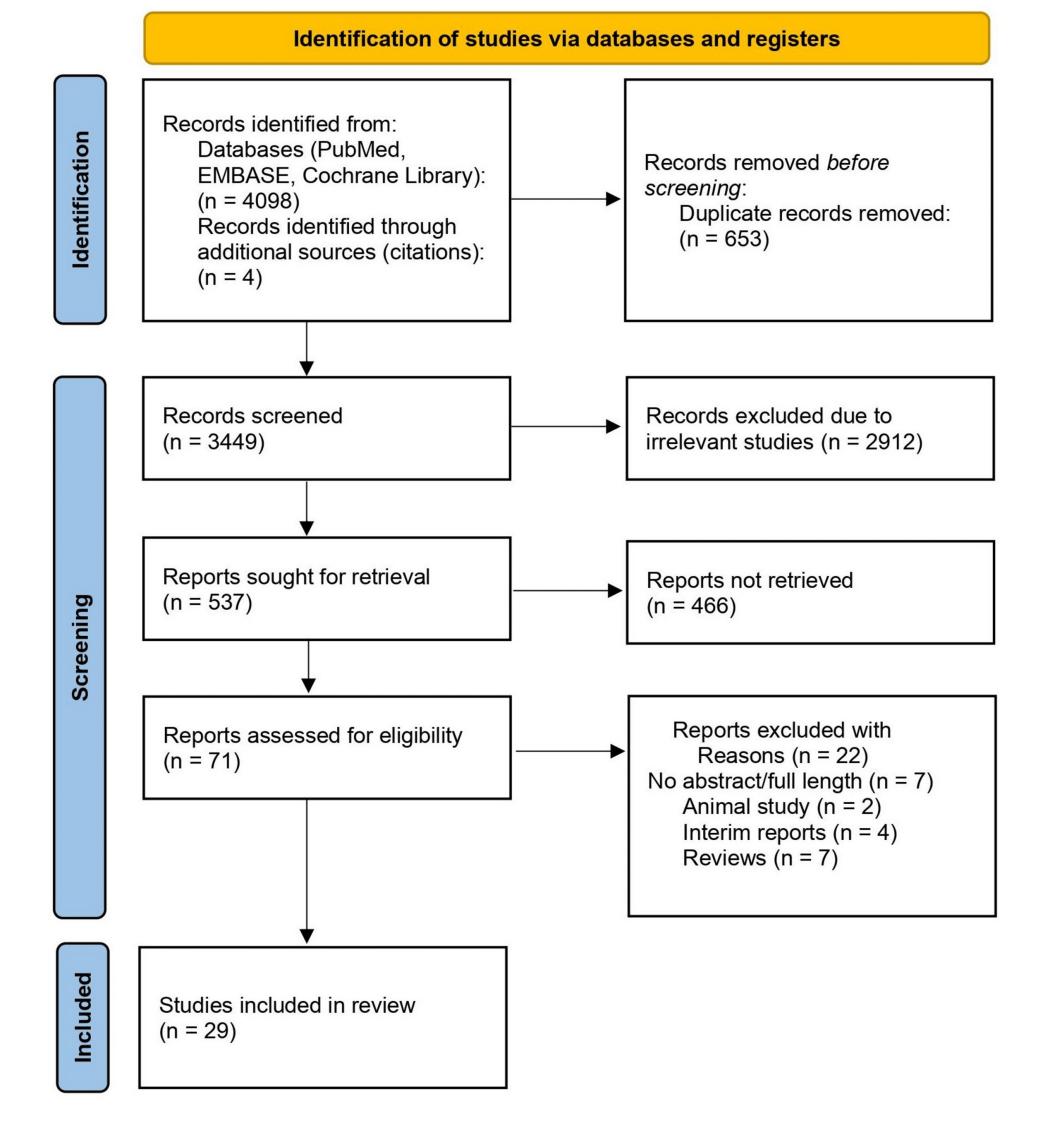
The Preferred Reporting Items for Systematic Reviews and Meta-Analyses flowchart.

We conducted a systemic and comprehensive literature search following the Preferred Reporting Items for Systematic Reviews and Meta-Analyses (PRISMA) protocol to ensure transparency in the search process (Figure 1) [13]. We conducted an initial search using major databases, including PubMed, Cochrane Library, and EMBASE. We used the Medical Subject Headings (MeSH) terms and keywords related to “COVID-19 vaccine,” “glomerulonephritis,” “p-ANCA,” and “MPO-ANCA” with the date of inception to March 5, 2024. We conducted our search without any language or geographical restrictions. Additionally, we also screened the references of included studies to identify all pertinent articles. We imported all citations to EndNote version 12.0 for comprehensive screening and extracting relevant data. Exclusion criteria involved studies non-relevant to COVID-19 vaccine-associated p-ANCA GN, review articles, editorials, and animal studies.

Based on the initial search, 4,102 articles were extracted, and two authors independently screened the titles and abstracts of the articles based on inclusion and exclusion criteria. Two authors assessed the eligibility of full-text articles of potentially relevant studies. Two independent authors evaluated the quality of the included studies based on the critical appraisal tool of the Joanna Briggs Institute for case studies. Relevant data, including study characteristics (author and publication year), patient demographics (age and sex), previous medical history, COVID-19 vaccine type, vaccine dose, time to onset of p-ANCA associated glomerulonephritis following vaccination, clinical presentation, laboratory findings, histopathological features, treatment modalities, and clinical outcomes was extracted from the eligible studies on pre-defined Microsoft Excel (Microsoft Corp., Redmond, WA, USA) datasheet. Data were extracted by two independent authors, and SPSS version 23.0 (IBM Corp., Armonk, NY, USA) was applied to demonstrate the numerical data as mean and standard deviation (SD) and categorical data as proportions (%). Discrepancies were solved through discussion and mutual consensus. A Measurement Tool to Assess Systematic Reviews 2 tool was used to assess the quality of our study [14].

Results

Of the 4,102 articles, 71 were assessed for eligibility, and 29 articles, including case reports and case series, were included in the review, reporting 35 patients with COVID-19 vaccine-induced p-ANCA GN. Each study’s characteristics (author and publication year), patient demographics (age and sex), previous medical history, COVID-19 vaccine type, vaccine dose, time to onset of p-ANCA-associated GN post-vaccination, clinical presentation, laboratory findings, histopathological features, treatment modalities, and clinical outcomes are tabulated in Table 3 [8,10-12,15-39].

**Table 3:** Clinical characteristics of patients presenting with GN following COVID-19 vaccine. COVID-19 = coronavirus disease 2019; HTN = hypertension; DM = diabetes mellitus; NA = not available; SSC = systemic sclerosis; HF = heart failure; IHD = ischemic heart disease; OM = otitis media; M = male; F = female; AKI = acute kidney injury; GN = glomerulonephritis; AIN = acute interstitial nephritis; fGN = fibrous glomerulonephritis; ATN = acute tubular necrosis; NR: not reported; ANCA = antineutrophil cytoplasmic antibody; p-ANCA = perinuclear antineutrophil cytoplasmic antibody; MPO-ANCA = myeloperoxidase antineutrophil cytoplasmic antibody; PEX = plasma exchange Hematuria:  Grade 1+ (clear pink color), Grade 2+ (light red color), Grade 3+ (cherry color). Proteinuria: Grade 1+ (Dipstick 1+), Grade 2+ (Dipstick 2+), Grade 3+ (Dipstick 3+), Grade 4+ (Dipstick 4+).

Authors	Age/Sex	Medical history	Vaccine type	Vaccine dose	New case/Relapse	Onset (days)	Symptoms	Hematuria	Baseline creatinine	Peak serum creatinine (mg/dL)	Urine protein	Extrarenal involvement	Diagnosis on biopsy	ANCA serologies	Treatment	Kidney pathology	Outcome
Uddin et al. [10]	59/M	HTN, IHD	Pfizer	Second	New	17	Fever, malaise, arthralgia	1+	NA	3.5	2+	NA	Yes	p-ANCA	Methylprednisolone, rituximab	Glomerulosclerosis, interstitial scarring	Remission
Javadian et al. [11]	32/F	NA	Sinopharm	Second	New	17	Fever, malaise, myalgia, fatigue	2+	NA	3.5	2+	Interstitial pneumonia	No	p-ANCA	Tocilizumab, plasmapheresis	No	Remission
El Hasbani et al. [12]	47/F	NA	Pfizer	First	New	3	Flank pain, hematuria, edema	2+	0.8	2.1	1+	NA	Yes	p-ANCA	Methylprednisolone	Crescentic GN, diffuse sclerosis	Remission
Gen et al. [15]	82/F	NA	Moderna	Third	New	30	Fever, malaise, hematuria, proteinuria	1+	0.73	1.4	1+	NA	Yes	p-ANCA	Prednisolone	Crescentic GN, rapidly progressive GN	Remission
Mazza et al. [16]	71/M	IHD	Pfizer	Third	New	21	Fever, hematuria	1+	0.91	2.1	2+	NA	Yes	p-ANCA	Dialysis, rituximab, steroids	Crescentic GN	Remission
Yoshino et al. [17]	56/M	NA	Pfizer	Second	New	42	Vomiting, abdominal pain	2+	0.83	1.07	2+	Aortitis, neuropathy	Yes	p-ANCA	Methylprednisolone, cyclophosphamide	Fibrinoid necrosis, Crescentic GN	Remission
Ting et al. [18]	63/F	Asthma	Pfizer	First	New	27	Renopulmonary syndrome	1+	NA	7.02	3+	NA	Yes	p-ANCA/c-ANCA	Steroids, cyclophosphamide, plasmapheresis	Crescentic GN	Remission
	51/M	HTN, HF	Pfizer	First	New	21	Renopulmonary syndrome	2+	NA	31.4	1+	NA	Yes	p-ANCA/c-ANCA	Steroids, cyclophosphamide, plasmapheresis	Crescentic GN	Remission with relapse
	62/F	NA	Pfizer	First	New	28	Renopulmonary syndrome	1+	NA	7.3	1+	NA	Yes	p-ANCA/c-ANCA	Steroids, cyclophosphamide, plasmapheresis	Segmental GN	Remission with Relapse
	70/M	HTN, DM, IHD	Moderna	Second	New	42	AKI	1+	NA	6.7	3+	NA	Yes	p-ANCA/c-ANCA	Steroids, cyclophosphamide, plasmapheresis	Fibrinoid necrosis, AIN	Remission
Kawamura et al. [19]	71/F	NA	Pfizer	Third	New	19	Fever, malaise, AKI	1+	0.71	3.5	2+	Interstitial pneumonia	Yes	p-ANCA	Steroids	AIN, fibrinoid necrosis	Remission
Baier et al. [20]	57/F	NA	Pfizer	Third	New	5	Dyspnea, hemoptysis	NA	NA	NA	NA	Pulmonary hemorrhage	No	p-ANCA	Steroids	NR	Remission
Chen et al. [21]	70/F	NA	Moderna	First	New	21	Hematuria, hemoptysis, AKI	3+	1	3.5	3+	Bilateral peri bronchial consolidation	Yes	p-ANCA	Steroids, rituximab, plasmapheresis	Crescentic GN, vasculitis	Remission
Cai et al. [22]	54/F	Polyarthritis, OM	Pfizer	Second	New	14	Constitutional symptoms, Episcleritis, edema	4+	NA	2.1	4+	Eye involvement, discreet opacities in both lungs	Yes	p-ANCA	Steroids, rituximab	Crescentic GN	Remission
Dube et al. [23]	29/F	CF	Pfizer	Second	New	16	AKI, nephritis	3+	0.8	1.9	NA	No	Yes	p-ANCA	Steroids, cyclophosphamide	Crescentic GN, fibrocellular crescentic GN, glomerulosclerosis, mild AIN	Remission
Hakroush et al. [24]	79/F	HTN	Pfizer	Second	New	14	Weakness, Thigh pain	2+	0.7	6.6	4+	Rhabdomyolysis	Yes	p-ANCA	Steroids, cyclophosphamide	Crescentic GN, glomerulosclerosis, ATN, AIN	Remission
Schaubshlager et al. [25]	77/F	HTN	Moderna	Second	New	84	AKI	NA	0.9	2.9	NA	No	Yes	p-ANCA	Steroids, Rituximab	Crescentic GN, fibrocellular crescentic GN, glomerulosclerosis, fibrinoid necrosis	Remission
Shakoor et al. [26]	78/F	HTN	Pfizer	Second	New/Follow-up	12	Nausea, vomiting, diarrhea	3+	0.8	1.3/3.5	2+	No	No	p-ANCA	No treatment/Steroids, rituximab	Crescentic GN, fibrinoid necrosis, AIN	Remission with relapse
Villa et al. [27]	63/M	NA	AstraZeneca	First	New	7	Hemoptysis, rapidly progressive GN	2+	Normal	2.9	2+	Interstitial pneumonia	Yes	p-ANCA	Steroids, cyclophosphamide	Crescentic GN	Remission
David et al. [28]	75/M	NA	AstraZeneca	First	Follow-up	35	Hemoptysis	3+	2.6	7	3+	Pulmonary hemorrhage	Yes	p-ANCA	Steroids, rituximab, Hemodialysis	Crescentic GN	Dialysis
	74/M	NA	AstraZeneca	Second	New	14	AKI	NA	0.9	10	NA	No	Yes	p-ANCA	Steroids, cyclophosphamide, hemodialysis	Crescentic GN with vasculitis	Remission
Sekar et al. [29]	47/F	NA	Pfizer	First	New	3	Flank pain, weakness	3+	0.8	2.9	NA	No	Yes	p-ANCA	Steroids, Azathioprine	fGN	Remission
Kim et al. [30]	72/F	NA	Moderna	Third	New	18	Constitutional symptoms, AKI	NA	0.8	4.7	+3	Otologic symptoms, gastritis	Yes	p-ANCA	Steroids, cyclophosphamide	fibrocellular crescentic GN, glomerulosclerosis, AIN	Remission
Loo et al. [31]	75/F	HF	Moderna	First	New	77	AKI	NA	NA	6.3	2+	No	Yes	p-ANCA	Steroids, rituximab, hemodialysis	Crescentic GN	Remission
Ma et al. [32]	70/F	HTN, dyslipidemia	CoronaVac	First	New	10	Constitutional symptoms, foamy urine	NA	0.5	5.4	2+	No	Yes	p-ANCA	Steroids, cyclophosphamide	Crescentic GN, fibrocellular crescentic GN, fGN	Remission
Noel et al. [33]	62/F	SSC, HTN, DM	Pfizer	Second	New	28	AKI	3+	1.0	5.2	NA	No	Yes	p-ANCA	Steroids, cyclophosphamide, mycophenolate mofetil	Crescentic GN, fibrocellular crescentic GN	Remission
Obata et al. [34]	84/M	Stroke	Pfizer	Second	New	14	Constitutional symptoms, cough	3+	1.2	1.2	1+	Interstitial pneumonia	Yes	p-ANCA	Steroids	Crescentic GN, glomerulosclerosis, fibrinoid necrosis	Remission
Ramezanzade et al. [35]	15/M	NA	Sinopharm	Second	New/follow-up	30	Constitutional symptoms, flank pain, nausea	4+	NA	3	3+	No	Yes	p-ANCA	Steroids, mycophenolate mofetil	Crescentic GN, necrotizing GN, ATN	Remission with relapse
So et al. [36]	42/M	NA	Pfizer	Second	New	63	Constitutional symptoms, gross hematuria	NA	1.0	3.1	4+	No	Yes	p-ANCA	Steroids, rituximab, PEX	Crescentic GN, fGN, mild interstitial infiltration	Remission
Suzuki et al. [37]	72/M	Prostatic hypertrophy	Pfizer	Second	New	1	Constitutional symptoms, AKI	3+	Normal	7.4	1+	No	Yes	p-ANCA	Steroids, rituximab, hemodialysis	Crescentic GN, segmental necrosis, fibrinoid necrosis	Remission
Zamoner et al. [38]	58/F	Hypothyroidism	AstraZeneca	First	New	5	Constitutional symptoms, foamy urine, arthralgia	1+	NA	2.2	4+	No	Yes	p-ANCA	Steroids, cyclophosphamide, azathioprine	crescentic GN, fibrocellular crescentic GN, fGN	Remission
Bansal et al. [39]	67/F	HTN	Covaxin/BBV152	Second	New	14	Edema, nausea, vomiting, decreased appetite	NA	1.2	6.4	3+	No	Yes	p-ANCA	Steroids, cyclophosphamide, hemodialysis	Fibrinoid necrosis, focal GN, crescentic GN	Remission
Thammathiwat et al. [8]	76/F	DM, HTN, dyslipidemia	AstraZeneca	Second	New	20	Fever, weight loss, cough, edema	2+	1.5	3.5	2+	Interstitial pneumonia	Yes	p-ANCA	Steroids, cyclophosphamide, Hemodialysis	Glomerulosclerosis, crescentic GN	Dialysis
	69/F	HTN	Pfizer	Third	New	28	Constitutional symptoms, AKI, hearing loss	1+	0.7	7.1	4+	Sensorineural hearing loss	Yes	p-ANCA	Steroids, cyclophosphamide, plasmapheresis	AIN, crescentic GN	Remission
	84/F	HTN, dyslipidemia	Moderna	Third	New	30	Constitutional symptoms, AKI,	1+	0.9	5.1	3+	Optic neuritis, valerian degeneration	Yes	p-ANCA	Steroids, IV immunoglobulin, hemodialysis	AIN, Fibrinoid necrosis, crescentic GN	Remission

In our study, 23 (65.7%) were females, and 12 (34.3%) patients were male with a median age of 69 years (mean ± SD = 63.22 ± 16). Thirteen patients reported hypertension as a previous medical history, and two patients had a history of kidney disease on initial presentation (Table 4).

**Table 4:** Demographics of study participants. N = number of patients; SD = standard deviation

Characteristics	Mean	SD
Age (years)	63.22	16.00
Sex	N	%
Male	12	34.3%
Female	23	65.7%
Past medical history		
Hypertension	13	37.14%
Diabetes mellitus	3	8.6%
Kidney disease	2	5.7%
No history/Not reported	15	42.9%

Our study also revealed that among the patients with COVID-19 vaccine-induced p-ANCA GN, 19 (54.28%) received Pfizer, and seven (20%) received the Moderna vaccine. Twenty-six (74.28%) patients received the mRNA vaccine (Pfizer plus Moderna), and four (11.42%) patients received the inactivated COVID-19 vaccine. Regardless of the type of the COVID-19 vaccine, 17 (48.57%) patients presented with p-ANCA GN after the second dose of the COVID-19 vaccine, and 11 (31.43%) patients presented after the first dose of the COVID-19 vaccine. Most cases presented with a median gap of 19 days (range = 1-84), and the majority of the patients (82.86%) presented after seven days of receiving the COVID-19 vaccine (Table 5).

**Table 5:** COVID-19 vaccine types and their percentage. N = number of patients; COVID-19 = coronavirus disease 2019; mRNA = messenger ribonucleic acid

Vaccine type	N	%
Pfizer	19	54.28
Moderna	7	20
AstraZeneca	5	14.28
Sinopharm	2	5.7
CoronaVac	1	2.9
Covaxin	1	2.9
mRNA vaccine	26	74.28
Inactivated vaccine	4	11.42
Adenovirus vector vaccine	5	14.28
Vaccine dose		
First	11	31.43
Second	17	48.57
Third	7	20
Days to first symptom onset		
Day <2	1	5.7
Day 2–7	5	14.28
Day >7	29	82.86

Constitutional symptoms (fever, malaise, myalgia, nausea, and vomiting) were the most reported manifestations in patients with vaccine-induced p-ANCA GN (54.28%), followed by AKI (42.85%), with 15 (42.85%) patients presented with extrarenal manifestations of p-ANCA (Table 6). Among laboratory findings, the mean baseline serum creatinine was 0.96 ± 0.39 mg/dL, and the mean peak serum creatinine was 4.98 ± 5.02 mg/dL. Hematuria was present in 27 (77.14%) patients, and protein urea was present in 29 (82.85%) patients (Table 7).

**Table 6:** Initial manifestations of patients with glomerulonephritis. N = number of patients; ENT = ear nose throat

Symptoms	N	%
Acute kidney injury	15	42.85
Constitutional symptoms	19	54.28
Edema	3	8.52
Hemoptysis	4	11.42
Renopulmonary syndrome	3	8.52
Extrarenal manifestations		
Lung (interstitial pneumonia, pulmonary hemorrhage)	7	20
Gastrointestinal	1	2.86
Eye	2	5.71
Neuromuscular	3	8.57
ENT	2	5.71

**Table 7:** Renal function parameter of patients with glomerulonephritis. SD = standard deviation; N = number of patients

Renal function test parameter		
Serum creatinine (mg/dL)		
Mean baseline	0.96; SD = ±0.39	
Mean peak	4.98; SD = ±5.02	
	N	%
Proteinuria		
Grade ≤2	16	45.71
Grade >2	13	37.14
Hematuria		
Grade ≤2	17	48.57
Grade >2	10	28.57

On serological testing, MPO/p-ANCA was positive in 31 (88.6%) patients, and dual-positive ANCA was detected in four (11.4%) patients. All patients were diagnosed with biopsy, and crescentic GN was the most common finding reported in 27 (77.14%) patients, followed by fibrinoid necrosis (25.71) (Table 8).

**Table 8:** Antibody serology and renal histopathology findings of the study participants. GN = glomerulonephritis; N = number of patients; MPO/p = myeloperoxidase/perinuclear; ANCA = antineutrophil cytoplasmic antibody

ANCA serologies	N	%
MPO/ANCA (p-ANCA)	31	88.6
p-ANCA + c-ANCA (dual positive)	4	11.4
Histopathology findings		
Crescentic GN	27	77.14
Fibrocellular crescentic GN	6	17.14
Glomerulosclerosis	8	22.86
Acute interstitial nephritis	8	22.86
Fibrinoid necroses	9	25.71

All patients were managed in accordance with the Kidney Disease Improving Global Outcomes 2021 guidelines for the management of glomerular diseases [41]. Steroids were prescribed in 30 (85.71%) patients, followed by rituximab (28.57%) and plasmapheresis (22.86%). Most patients responded well to treatment, and 29 (82.86%) patients achieved complete remission and relapse in four (11.42%) patients. Two patients were hemodialysis-dependent in non-remission, with no mortality reported (Table 9).

**Table 9:** Advised treatment and treatment outcomes of the study participants. N = number of patients

Used treatment	N	%
Steroids	30	85.71
Rituximab	10	28.57
Plasmapheresis	8	22.86
Dialysis	10	28.57
Treatment outcomes		
Remission	29	82.86
Remission with relapse	4	11.42
Maintenance dialysis	2	5.72

Discussion

Considering the ongoing emerging data between the COVID-19 vaccine and kidney diseases, public concerns have risen regarding the potential kidney adverse events. COVID-19 vaccine-associated renal complications include AKI, acute interstitial nephritis, minimal change disease, renal vasculitis, immunoglobulin A nephropathy, and membranous nephropathy [7]. p-ANCA GN is a life-threatening complication of the COVID-19 vaccine, which is not widely reported [14]. A systematic review of renal side effects of the COVID-19 vaccine reported only 16 patients of ANCA out of 128 patients. Seven of the 16 patients were diagnosed with p-ANCA GN triggered by the COVID-19 vaccine. Most patients presented with constitutional symptoms and acute kidney disease after the second dose of the COVID-19 vaccine, with a median onset time of 14 days, consistent with our study’s result [7]. Campos et al. [40] reported 11 cases of p-ANCA GN following the COVID-19 vaccine, and mRNA vaccines were the main culprits in triggering GN. Of the 11 cases, nine were female, and the patients presented with non-specific symptoms with an average of 18 days of the vaccine. All patients responded to treatment except for two who remained on dialysis [41]. Thammathiwat et al. reported a case series of three patients who presented with non-specific symptoms and AKI following the COVID-19 vaccine. After laboratory evaluations and biopsy findings, the patients were diagnosed with p-ANCA GN induced by the COVID-19 vaccine. Immunosuppressant therapy and plasmapheresis resulted in complete remission [8].

Our study revealed that COVID-19 vaccine-induced p-ANCA GN was predominant in females with a median age of 69 years, and hypertension was the most common comorbidity among these patients, consistent with published data and highlighting the importance of considering underlying medical conditions in the context of vaccine-related side effects. Our study also highlighted that the Pfizer vaccine was most frequently implicated in p-ANCA GN, and the majority of cases occurred after the second dose of the COVID-19 vaccine, consistent with other studies. This temporal association underscores the need to closely monitor patients following vaccination and promptly evaluate any concerning symptoms. Clinical presentations and laboratory findings revealed characteristic features of renal involvement, including elevated renal function tests, hematuria, and proteinuria. Serological testing demonstrated positivity for p-ANCA in patients, suggesting an autoimmune etiology of the disease, and histopathological examination of renal biopsies further supported the diagnosis of p-ANCA GN. Patients in our study were managed following the treatment guidelines for glomerular diseases, focusing on immunosuppressive therapy. Most patients responded well to treatment, with a high proportion achieving complete remission. Despite the favorable treatment response in the majority of patients, a subset of patients experienced relapse, emphasizing the extended monitoring and management of vaccine-induced p-ANCA GN [8].

COVID-19 vaccines, especially mRNA vaccines, have been linked to an increased prevalence of p-ANCA GN, mainly due to their high immunogenicity and global use [7,42]. Lipid-based nanoparticles of mRNA COVID-19 vaccines induce activation of CD4+ and CD8+ T-cell lymphocytes and enhance an increased production of B cells in the germinal centers, leading to increased secretion of inflammatory mediators, such as interferons and interleukin-2 (IL-2). DNA adenoviral vaccine induces CD4+ and CD8+ cytotoxic T cells and induces B-cell lymphocytes to enhance antibody production, mainly immunoglobulins G1-G4. Viral spike proteins have also been linked to glomerular injury [43].

Compared to drug-induced ANCA GN, COVID-19 vaccine-induced ANCA GN presents a remarkably shorter median onset, often occurring after second or booster doses of the COVID-19 vaccine, suggesting a potential booster effect, contributing to a higher absolute risk of GN [8]. The underlying mechanism of vaccine-induced ANCA GN remains undefined. It is postulated that antiviral-neutralizing antibodies may induce an autoimmune response, resulting in ANCA production [11,44]. Another mechanism may involve molecular mimicry activating B and T cells via Toll-like receptors and causing a robust immune response, potentially priming ANCA production. Furthermore, an innate immune response triggered by vaccine adjuvants such as factor MF59 in the AstraZeneca COVID-19 vaccine may also contribute to developing immune-mediated GN [9,45]. Additionally, immune response results in a cascade of inflammatory events by inflammatory cytokines, including IL-6, interferon-gamma, and the formation of neutrophil extracellular traps containing MPO and PR3, further mediating glomerular injury. The interplay between COVID-19 vaccine-induced ANCA GN and autoantibodies suggests a complex relationship that warrants further exploration [20].

Our study has many implications for public health policy, vaccination strategies, and risk communication. Continuous monitoring and reporting of vaccine-induced complications, including ANCA GN, are mandatory [4,13]. Our study also highlights the need for standardized diagnostic criteria to diagnose vaccine-induced ANCA GN and comprehensive management guidelines. Ongoing research endeavors are obligatory to uncover the pathological mechanisms of vaccine-induced ANCA GN, mainly mRNA vaccines triggering immune-mediated GN. Collaborative research initiatives, genetic analyses, inflammatory pathways, and thorough autoimmune response after a vaccine are required to unravel the complex interplay of this vaccine-induced rare adverse event.

Despite valuable implications, our systematic review has a few limitations, including generalizability due to small sample data, incomplete understating of pathophysiological mechanisms, and reporting bias. Our study has no control group, limiting the ability to establish direct causal relationships between ANCA GN and the COVID-19 vaccine. Additionally, most patients reported no history of COVID-19 infection or kidney disease. These limitations provide insights for further research to improve the understanding of COVID-19 vaccine-induced ANCA GN.

## Conclusions

COVID-19 vaccine-induced p-ANCA GN represents an uncommon but significant adverse event after vaccination. Our study provides valuable insights into the clinical characteristics, management, and outcomes of COVID-19 vaccine-induced p-ANCA GN. Timely recognition, probable diagnosis, and effective management are crucial in optimizing patient outcomes and ensuring effective surveillance. Physicians should remain vigilant regarding potential renal complications of the COVID-19 vaccine, particularly p-ANCA GN, and it should be included in the differential diagnosis of patients presenting with non-specific symptoms and AKI after the vaccine, mainly in the context of mRNA COVID-19 vaccine. Further research is warranted to unravel the underlying pathophysiology of COVID-19 vaccine-induced p-ANCA GN.
