# Correction: A Systematic Review of Perinuclear Antineutrophil Cytoplasmic Antibody-Associated Glomerulonephritis Following Coronavirus Disease 2019 Vaccination: A 2024 Update

**DOI:** 10.7759/cureus.c339

**Published:** 2025-10-01

**Authors:** Ikponmwosa J Ogieuhi, FNU Suman, Nikita Kumari, Bai Manita, Dinkey Kumari, Joti Devi, Mohamed Abdalla, Eithar Shabbo, Utsav Patel, Iqra Samreen, Khalid H Mohamed, Zahoor Ahmed, Hira Nasir

**Affiliations:** 1 Physiology, University of Benin, Benin City, NGA; 2 General Medicine, Siberian State Medical University, Tomsk, RUS; 3 Internal Medicine, Ghulam Muhammad Mahar Medical College, Sukkur, PAK; 4 Internal Medicine, Peoples University of Medical and Health Sciences, Nawabshah, PAK; 5 Pharmacy, Clifton Medical Services, Karachi, PAK; 6 Internal Medicine, Dallah Hospital, Riyadh, SAU; 7 School of Medicine, Ahfad University for Women, Omdurman, SDN; 8 Internal Medicine, Medical College, Baroda and Sir Sayaji General (SSG) Hospital, Vadodara, IND; 9 Medical School, Deccan College of Medical Sciences, Hyderabad, IND; 10 Neurology, Sheffield Teaching Hospitals NHS Foundation Trust, Sheffield, GBR; 11 Internal Medicine, Mayo Hospital, Lahore, PAK

This article has been corrected to fix the following typo in the first paragraph of the Results section: "...48 were assessed for eligibility" has been corrected to "...71 were assessed for eligibility."

